# Homocysteine regulates fatty acid and lipid metabolism in yeast

**DOI:** 10.1074/jbc.M117.809236

**Published:** 2018-02-06

**Authors:** Myriam Visram, Maja Radulovic, Sabine Steiner, Nermina Malanovic, Thomas O. Eichmann, Heimo Wolinski, Gerald N. Rechberger, Oksana Tehlivets

**Affiliations:** From the ‡ Institute of Molecular Biosciences, University of Graz, Humboldtstrasse 50, 8010 Graz, Austria and; the § Omics Center Graz, BioTechMed-Graz, 8010 Graz, Austria

**Keywords:** fatty acid, homocysteine, lipid, Saccharomyces cerevisiae, triacylglycerol, S-adenosyl-L-homocysteine, S-adenosyl-L-homocysteine hydrolase, hyperhomocysteinemia

## Abstract

*S*-Adenosyl-l-homocysteine hydrolase (AdoHcy hydrolase; Sah1 in yeast/AHCY in mammals) degrades AdoHcy, a by-product and strong product inhibitor of *S*-adenosyl-l-methionine (AdoMet)-dependent methylation reactions, to adenosine and homocysteine (Hcy). This reaction is reversible, so any elevation of Hcy levels, such as in hyperhomocysteinemia (HHcy), drives the formation of AdoHcy, with detrimental consequences for cellular methylation reactions. HHcy, a pathological condition linked to cardiovascular and neurological disorders, as well as fatty liver among others, is associated with a deregulation of lipid metabolism. Here, we developed a yeast model of HHcy to identify mechanisms that dysregulate lipid metabolism. Hcy supplementation to wildtype cells up-regulated cellular fatty acid and triacylglycerol content and induced a shift in fatty acid composition, similar to changes observed in mutants lacking Sah1. Expression of the irreversible bacterial pathway for AdoHcy degradation in yeast allowed us to dissect the impact of AdoHcy accumulation on lipid metabolism from the impact of elevated Hcy. Expression of this pathway fully suppressed the growth deficit of *sah1* mutants as well as the deregulation of lipid metabolism in both the *sah1* mutant and Hcy-exposed wildtype, showing that AdoHcy accumulation mediates the deregulation of lipid metabolism in response to elevated Hcy in yeast. Furthermore, Hcy supplementation in yeast led to increased resistance to cerulenin, an inhibitor of fatty acid synthase, as well as to a concomitant decline of condensing enzymes involved in very long-chain fatty acid synthesis, in line with the observed shift in fatty acid content and composition.

## Introduction

**Figure 1.**
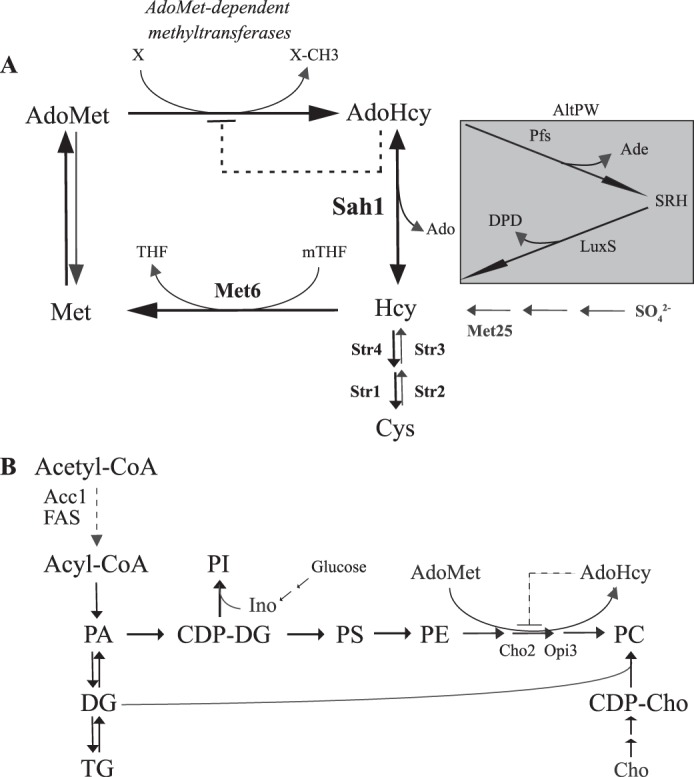
**Methylation and glycerolipid metabolism in yeast.** *A*, Sah1 catalyzes the reversible hydrolysis of AdoHcy to Hcy and adenosine. *B*, an accumulation of AdoHcy inhibits PC synthesis from PE by the three-step AdoMet-dependent methylation pathway. The alternative bacterial pathway for AdoHcy catabolism (AltPW) is shown in the *gray box* in *A*.

Methylation plays a pivotal role in cellular metabolism. These reactions, catalyzed by more than 200 different methyltransferases in mammals (1), depend on *S*-adenosyl-l-methionine (AdoMet)^2^ as a methyl donor and generate *S*-adenosyl-l-homocysteine (AdoHcy) as a by-product (2). AdoHcy is a strong product inhibitor of AdoMet-dependent methyltransferases (2). *S*-Adenosyl-l-homocysteine hydrolase (Sah1 in yeast/AHCY in mammals) is the sole enzyme responsible for the degradation of AdoHcy to adenosine and homocysteine (Hcy) (Fig. 1*A*) (2). Deletion of the locus overlapping the *AHCY* gene in mice leads to embryonic lethality (3). Deletion of the *SAH1* gene in yeast results in drastically reduced growth (4). Growth of the *sah1* mutants is fully abolished by additional deletion of the *MET25* gene (or any other gene) in the “sulfur assimilation pathway” (4). This pathway, which is absent in mammals, allows yeast to synthesize Hcy from sulfate in the growth medium. Hcy synthesized in the course of methylation metabolism or by the sulfur assimilation pathway can be remethylated to methionine, which can be further activated to AdoMet, or converted to cysteine, a precursor of glutathione (Fig. 1*A*) (5). Thus, Sah1/AHCY not only plays an important role in regulating methylation metabolism by degrading AdoHcy, but in mammals it also constitutes the sole pathway for the synthesis of Hcy, which is typically absent from the diet (5).

A pathological condition in humans termed hyperhomocysteinemia (HHcy) is characterized by elevated levels of Hcy in the serum (6). HHcy is linked to a number of disorders of modern society, including cardiovascular and neurological diseases, fatty liver, insulin resistance, and cancer (6–9). Notably, HHcy is present in 5–10% of the general population (10) and up to 30% of the elderly (>65 years) (11); however, mechanisms linking Hcy accumulation to disease phenotypes are unclear. Because the Sah1/AHCY-catalyzed reaction is reversible and the equilibrium of the reaction *in vivo* strongly favors the anabolic direction (4, 12–14), any elevation of Hcy levels, such as in HHcy, drives the formation of AdoHcy, which was shown to be a more sensitive marker of HHcy-associated pathology than Hcy (15, 16) .

Elevated Hcy and/or AdoHcy are associated with triacylglycerol (TG) accumulation in liver, endothelial, and smooth muscle cells (7, 17–19), adipocyte dysfunction (20, 21), and overall loss of fat mass (22), indicating a major impact on cellular lipid homeostasis in mammals. Furthermore, both elevated Hcy and AdoHcy lead to a depletion of polyunsaturated fatty acids in liver and plasma phospholipids (23–26). Some of these phenotypes are consistent with an inhibition of phospholipid (PL) methylation, which is also associated with TG accumulation in the liver and polyunsaturated fatty acid depletion (27–29). In mammals, about 50% of total AdoMet is used for the *de novo* synthesis of the major membrane PL, phosphatidylcholine (PC), by the three-step AdoMet-dependent methylation of phosphatidylethanolamine (PE) (Fig. 1*B*) (30, 31). PL methyltransferases that catalyze PC synthesis by the methylation pathway in mammals as well as in yeast are particularly sensitive to AdoHcy *in vitro* (32, 33).

Sah1/AHCY (EC 3.3.1.1) is an exceptionally well-conserved enzyme that exhibits 70% sequence identity at the protein level between human and yeast orthologs (34). Because degradation of AdoHcy is a universal regulator of methylation metabolism, some organisms, including *Escherichia coli*, that lack Sah1/AHCY orthologs, have evolved an alternative irreversible two-step pathway for AdoHcy catabolism that involves *S*-adenosylhomocysteine nucleosidase (Pfs, EC 3.2.2.9) and *S*-ribosylhomocysteine lyase (LuxS, EC 4.4.1.21) (35) (Fig. 1*A*). This pathway is referred to as AltPW (alternative pathway) throughout this study. We have previously shown that Sah1 is regulated at the transcriptional level in coordination with PL biosynthetic genes; *SAH1* expression is repressed in the presence and derepressed in the absence of lipid precursors, inositol and choline (36). Similarly to Hcy supplementation, down-regulation of *SAH1* expression leads to AdoHcy and TG accumulation in yeast in the absence of inositol and choline (4). Here, we dissected AdoHcy- from Hcy-evoked effects by expressing the alternative irreversible bacterial pathway for AdoHcy degradation in wildtype yeast and *sah1* mutants. The AltPW indeed fully suppressed the growth defect of the *sah1* mutant, providing clear evidence that the poor growth of the *sah1* mutant is linked to AdoHcy accumulation. In addition, the AltPW suppressed Hcy-induced lipid alterations, including TG accumulation, increased FA content, and altered FA profiles, showing that indeed AdoHcy accumulation is the key trigger of the deregulation of lipid metabolism in response to Hcy in yeast. This deregulation appears to involve the deregulation of the enzymes involved in FA synthesis, FA synthase (FAS) as well as condensing enzymes of FA elongase complex and FA desaturase, by AdoHcy, contributing to the complex alterations in cellular lipid homeostasis.

## Results

### The growth defects of the sah1 single and sah1met25 double mutants are of different nature

**Figure 2.**
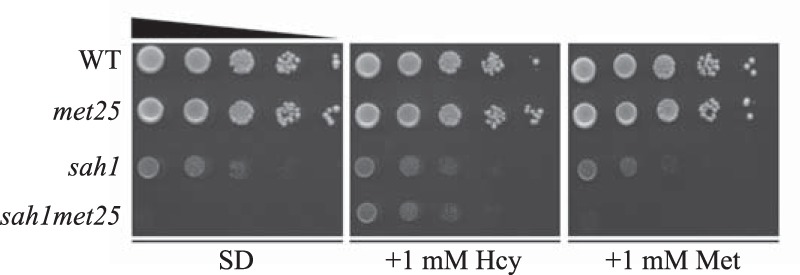
**The growth defect of the yeast *sah1met25* but not *sah1* mutant is rescued by Hcy supplementation.** Wildtype yeast and the *met25*, *sah1*, and *sah1met25* mutants were analyzed for growth on SD medium containing or not containing 1 mm Hcy and 1 mm Met. Images were taken after 2 days of growth at 30 °C.

Sah1/AHCY offers a single way to dissipate the potent product inhibitor AdoHcy in yeast and mammals and also provides Hcy for cysteine synthesis and/or remethylation to methionine. The yeast *sah1* mutant is viable, displaying markedly impaired growth; additional disruption of the sulfur assimilation pathway in the *sah1* mutant leads, however, to inviability of the resulting double mutant, unless it is cultivated in the presence of Hcy (4) (Fig. 2). Because Hcy can be used for cysteine/glutathione or methionine/AdoMet synthesis, we wanted to investigate blockage of which pathway is responsible for the inviability of the *sah1met25* double mutant. Supplementation of 1 mm Hcy suppressed the growth defect of the *sah1met25* but not of the *sah1* mutant (Fig. 2). In contrast, the addition of 1 mm methionine to the standard growth medium (which already contains ∼0.1 mm methionine) did not improve growth of either the *sah1met25* or *sah1* mutant (Fig. 2). This indicates that inviability of the *sah1met25* double mutant is due to a block in Hcy synthesis, presumably leading to glutathione depletion and oxidative stress induction. In contrast, the growth defect of the *sah1* mutant is instead due to AdoHcy accumulation (Fig. 2).

### Wildtype cells exposed to Hcy show similar alterations in lipid composition as the sah1 mutant

**Figure 3.**
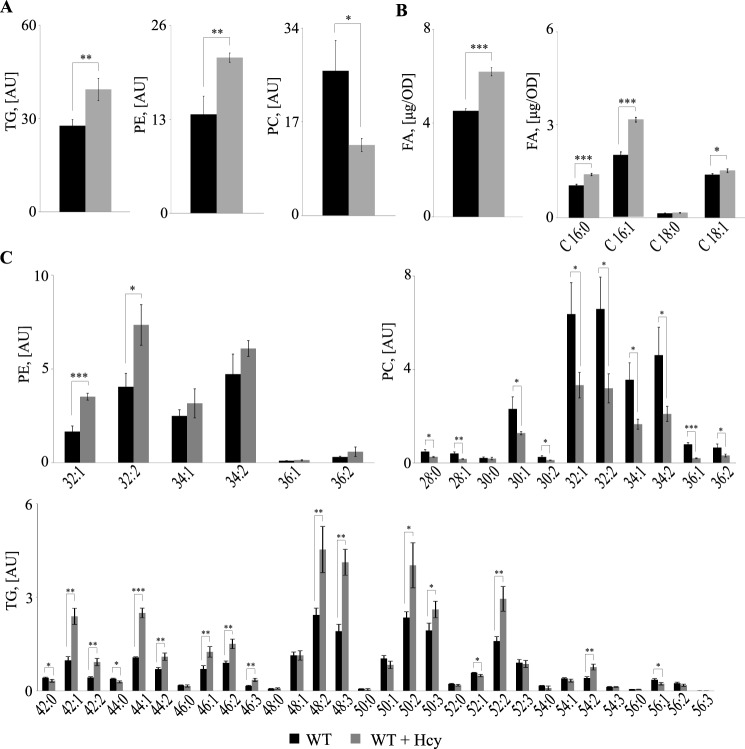
**Hcy-supplemented wildtype yeast accumulate FAs and exhibit altered FA composition in the main glycerolipids.** *A*, total TG, PE, and PC levels. *B*, total FA levels and total FA composition. *C*, PE, PC, and TG species distribution. Logarithmic-phase wildtype cells were grown overnight in +I/+C, shifted to −I/−C containing or not containing 1 mm Hcy, and cultivated for 6 h before lipid extraction. TG, PE, PC, and FA were normalized to ISTDs. Data are mean ± S.D. (*error bars*) from three independent experiments (***, *p* < 0.001; **, *p* < 0.01; *, *p* < 0.05).

**Figure 4.**
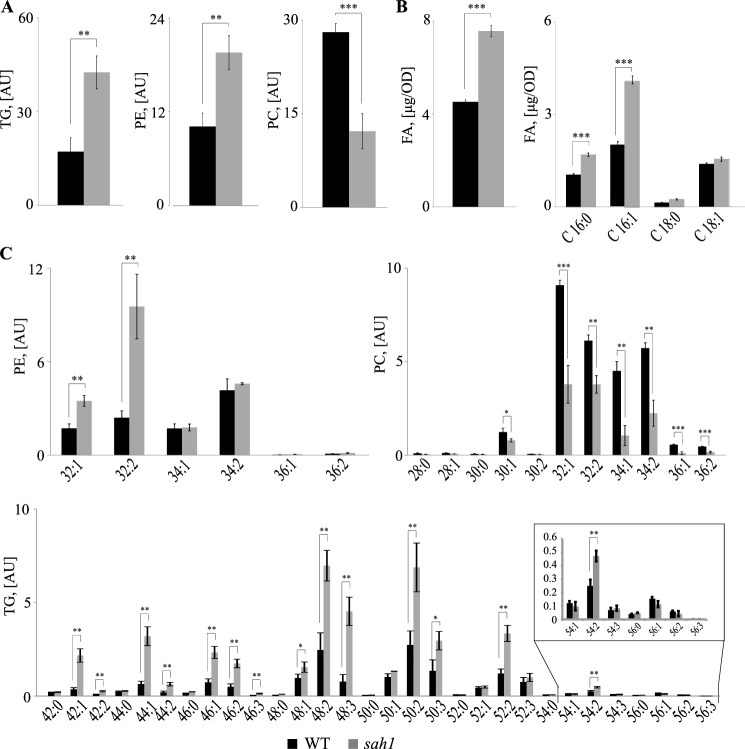
**The yeast *sah1* mutant phenocopies the deregulation of lipid metabolism in Hcy-supplemented wildtype.** *A*, total TG, PE, and PC levels. *B*, total FA levels and total FA composition. *C*, PE, PC, and TG species distribution. Logarithmic-phase wildtype and the *sah1* mutant yeast cells were grown overnight in +I/+C, shifted to −I/−C, and cultivated for 6 h before lipid extraction. TG, PE, PC, and FA were normalized to ISTDs. Data are mean ± S.D. (*error bars*) from three independent experiments (***, *p* < 0.001; **, *p* < 0.01; *, *p* < 0.05).

We have previously shown that down-regulation of *SAH1* expression in yeast leads to a concomitant increase of AdoHcy and TG content in the absence of inositol and choline (4). To simulate conditions of HHcy in our yeast model, wildtype cells were supplemented with 1 mm Hcy and analyzed for lipid composition. In accordance with our previous observations, Hcy-supplemented wildtype yeast cultivated in the absence of inositol and choline (−I/−C) exhibited elevated TG levels similarly to the *sah1* mutant grown in the absence of Hcy (Figs. 3*A* and 4*A*). Most notably, both Hcy-supplemented wildtype and the *sah1* mutant displayed elevated total FA content by some 30–60%, respectively (Figs. 3*B* and 4*B*). This indicates that the altered glycerolipid profiles in Hcy-supplemented wildtype yeast and the *sah1* mutant are not simply a result of the redirection of FA metabolic flux from PL toward TG due to inhibition of PE methylation and decreased PC synthesis via the CPD-choline pathway in the absence of choline (37) (Figs. 3*A* and 4*A*) but rather a result of an up-regulated *de novo* FA synthesis. Furthermore, analysis of total FA composition showed a selective increase in palmitoleic (C16:1) acid (Figs. 3*B* and 4*B*), which was also observed in glycerolipids and was most pronounced in PE and TG. PE32:2 was increased 2–4-fold, and TG48:3 was increased 2–5-fold in both Hcy-supplemented wildtype and the *sah1* mutant, respectively, as compared with control cells (Figs. 3*C* and 4*C*). This shift in FA profiles suggests an overall up-regulated production of FAs but also a decreased FA elongation and an increased FA desaturation in response to Hcy in yeast (Figs. 3*B* and 4*B*). Of note, Hcy supplementation did not impair wildtype growth (Fig. 2). This suggests that the increase in AdoHcy through the bidirectionally functional Sah1 reaction in Hcy-supplemented wildtype yeast may not induce the same detrimental consequences on cellular methylation as a complete loss of Sah1 activity.

### Expression of the alternative irreversible pathway for AdoHcy catabolism (AltPW) functionally complements Sah1 deficiency in yeast

**Figure 5.**
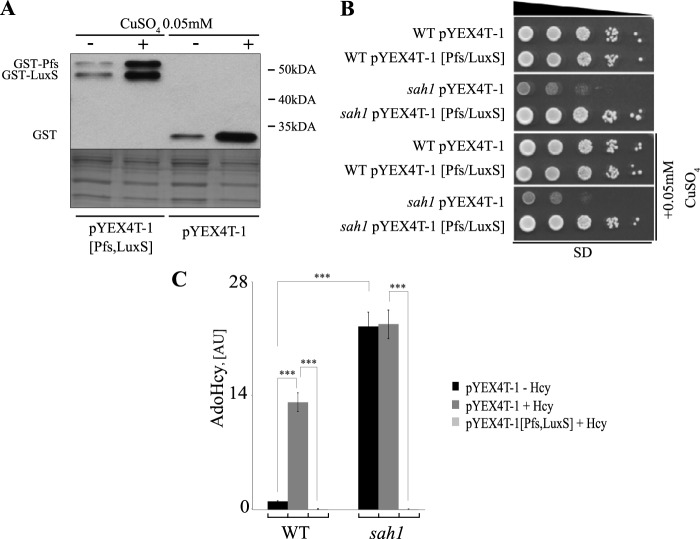
**The AltPW functionally complements the yeast *sah1* mutants.** *A*, expression of N-terminally tagged GST-Pfs and GST-LuxS fusion proteins in yeast. Shown are heterozygous diploid *sah1/SAH1 met25/MET25* transformants carrying the empty pYEX4T-1 or pYEX-4T-1 [Pfs/LuxS] plasmid containing *E. coli pfs* and *luxS* genes under the regulation of the *CUP1^p^* promoter. *B*, the AltPW suppresses the growth defect of the *sah1* mutant. Wildtype yeast and the *sah1* mutant carrying pYEX4T-1 or pYEX-4T-1 [Pfs/LuxS] plasmid were analyzed for growth on SD media with or without 0.05 mm CuSO_4_. Images were taken after 2 days of growth at 30 °C. *C*, the AltPW suppresses elevated AdoHcy levels in the *sah1* mutant and in Hcy-supplemented wildtype yeast. Logarithmic-phase wildtype and the *sah1* mutant yeast cells carrying pYEX4T-1 or pYEX-4T-1 [Pfs/LuxS] plasmid were cultivated in SD−Ura with or without 0.05 mm CuSO_4_ and 1 mm Hcy for 6 h before AdoHcy extraction. AdoHcy levels only for strains grown in the absence of additional CuSO_4_ supplementation are shown. AdoHcy was normalized to ISTD. Data are mean ± S.D. (*error bars*) from three independent experiments (***, *p* < 0.001).

Hcy supplementation leads not only to Hcy but also to AdoHcy accumulation (4). Thus, we set out to dissect AdoHcy- and Hcy-triggered effects on lipid metabolism in yeast by expressing the AltPW (Fig. 1*A*) in wildtype and *sah1* mutant cells. *pfs* and *luxS* genes encoding *S*-adenosylhomocysteine nucleosidase and *S*-ribosylhomocysteine lyase were cloned from *E. coli* wildtype cells into a yeast episomal plasmid as GST fusions, as described under “Experimental procedures.” Expression of both genes was under the control of the copper-inducible *CUP1^p^* promoter. As shown in Fig. 5*A*, both fusion proteins were expressed in yeast and yielded distinct bands with the corresponding sizes of 24 kDa for Pfs and 19 kDa for LuxS on a Western blot. Proteins were already detectable in the absence of additional copper supplementation, indicating that the traces of copper in the standard growth medium were already sufficient to drive Pfs and LuxS expression. The AltPW fully restored wildtype growth of the *sah1* mutant, suggesting that the expressed enzymes indeed degraded AdoHcy. Consistent with the Western blot data shown in Fig. 5*A*, growth complementation of the *sah1* mutant was already observed in the absence of copper supplementation (Fig. 5*B*), and the addition of 0.05 mm copper had no further effect on the growth. Because we proposed that growth inhibition of the *sah1* mutant was due to a buildup of AdoHcy, we next measured the content of this metabolite both in wildtype and in the *sah1* mutant transformed with the expression plasmid harboring the AltPW. As shown in Fig. 5*C*, wildtype cells transformed with the empty plasmid showed a massive accumulation of AdoHcy in the presence of Hcy in the growth medium, consistent with the reversal of the Sah1-catalyzed reaction. Expression of the AltPW completely abolished the AdoHcy buildup in wildtype cells in the presence of Hcy, further confirming that the AltPW was functional (Fig. 5*C*). In contrast, the *sah1* mutant transformed with the empty plasmid displayed highly elevated AdoHcy content, as expected, in the absence of Hcy in the growth medium; the further addition of Hcy had no influence on the AdoHcy levels (Fig. 5*C*). Expression of the AltPW completely dissipated the AdoHcy buildup in the mutant (Fig. 5*C*). These data clearly show that (i) Hcy supplementation drives AdoHcy accumulation in wildtype cells, (ii) AdoHcy accumulation in the *sah1* mutant is independent of Hcy supplementation to the medium, and (iii) functional expression of the unidirectional bacterial AltPW leads to complete degradation of AdoHcy that accumulates in wildtype in the presence of Hcy or in the *sah1* mutant and also fully restores cell growth in the absence of Sah1.

### AdoHcy is responsible for the deregulation of lipid metabolism in the sah1 mutant and Hcy-supplemented wildtype

**Figure 6.**
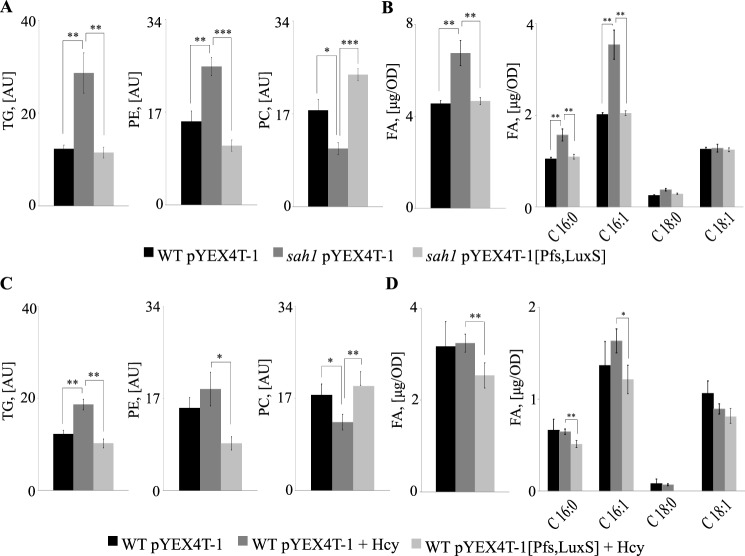
**Expression of the AltPW suppresses the deregulation of lipid metabolism in the *sah1* mutant and Hcy-supplemented wildtype yeast.** *A*, total TG, PE, and PC levels in the yeast *sah1* mutant. *B*, total FA levels and total FA composition in the yeast *sah1* mutant. *C*, total TG, PE, and PC levels in Hcy-supplemented wildtype yeast. *D*, total FA levels and total FA composition in Hcy-supplemented wildtype yeast. Logarithmic-phase wildtype and the *sah1* mutant yeast cells expressing the AltPW or carrying the empty plasmid were cultivated in SD−Ura (containing 10 μm inositol), reinoculated into the same medium with or without 0.05 mm CuSO_4_, and cultivated for 6 h before lipid extraction. Wildtype yeast expressing the AltPW or carrying the empty plasmid were cultivated in the presence or absence of 1 mm Hcy only in the SD−Ura without additional CuSO_4_ supplementation. Data for wildtype yeast and the *sah1* mutant grown in the presence of additional CuSO_4_ supplementation are not shown. TG, PE, PC, and FA were normalized to ISTDs. Data are mean ± S.D. (*error bars*) from three independent experiments (***, *p* < 0.001; **, *p* < 0.01; *, *p* < 0.05).

Hcy supplementation to wildtype yeast and lack of Sah1 activity both induce major alterations in cellular lipid homeostasis, presumably through a common mechanism of AdoHcy accumulation. Because expression of the AltPW fully suppressed AdoHcy accumulation in both strain backgrounds, we next set out to analyze its impact on cellular lipid content. Because the *sah1* mutant is an inositol-overproducing (*opi*) strain (4) and inositol may interfere with membrane lipid (38) and TG synthesis (39), we supplemented the culture medium with 10 μm inositol, which is present in standard yeast media (40). 10 μm inositol did not affect TG levels in the *sah1* mutant and Hcy-supplemented wildtype expressing the empty plasmid, suggesting that inositol is not involved in the deregulation of lipid metabolism in response to Hcy (Figs. 3, 4, and 6). In contrast, expression of the AltPW in the *sah1* mutant fully restored wildtype TG as well as FA content and composition (Fig. 6*A* and Fig. S1), showing that AdoHcy accumulation is indeed responsible for the deregulation of lipid metabolism in the *sah1* mutant. Notably, PE levels were somewhat decreased and PC levels increased in the presence of the AltPW, indicating very efficient removal of cellular AdoHcy that may otherwise inhibit PE-to-PC methylation. Although Hcy-induced lipid alterations in wildtype cells were less pronounced, in accordance with lower AdoHcy levels (Fig. 5), expression of the AltPW in Hcy-supplemented wildtype suppressed an accumulation of TG, restored its wildtype composition, and decreased the levels of total FA and in particular C16:1 (Fig. 6*B* and Fig. S2), indicating that AdoHcy indeed is the key trigger of the deregulation of lipid metabolism in response to Hcy in yeast.

### AdoHcy accumulation impacts FA synthesis at multiple levels

**Figure 7.**
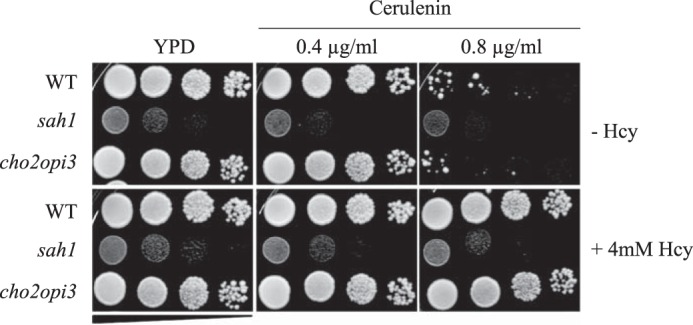
**Hcy-supplemented wildtype yeast as well as the *sah1* mutant are resistant to cerulenin.** Wildtype yeast and the yeast *sah1* and *cho2opi3* mutants were analyzed for growth on YPD containing or not containing 0.4–0.6 μg/ml cerulenin and 4 mm Hcy, as indicated. Images were taken after 2 days of growth at 30 °C.

A hallmark of AdoHcy accumulation both in the *sah1* mutant and in Hcy-supplemented wildtype was the increase in cellular FA and TG content and alterations in FA composition. Notably, these alterations reflect metabolic changes observed under conditions of HHcy in mammals (7, 8, 18, 19, 23–26). To obtain further insight into potential mechanisms underlying these alterations, we analyzed the sensitivity of Hcy-supplemented wildtype and the *sah1* mutant to the FAS inhibitor, cerulenin, which provides a reliable readout for *in vivo* activity of this enzyme (41). In addition, abundance and subcellular localization of FAS subunits Fas1 and Fas2 as well as condensing enzymes Elo1, Elo2, and Elo3 of the FA elongation complex and Ole1, the sole FA desaturase in yeast, were determined by Western blotting and live cell fluorescence microscopy of C-terminal GFP fusions, respectively. Markedly increased resistance of Hcy-supplemented wildtype to cerulenin suggests increased FAS activity (Fig. 7). Although the *sah1* mutant displayed overall reduced growth under all conditions, it did not appear to show any response to the inhibitor in the absence or presence of Hcy (Fig. 7). Because this mutant accumulates AdoHcy under all conditions, this finding suggests that cerulenin resistance is linked to AdoHcy content. In contrast, the *cho2opi3* mutant, deficient in PL methylation without being impaired in cellular methylation due to AdoHcy accumulation, exhibited wildtype sensitivity to cerulenin in the absence or presence of Hcy (Fig. 7). This shows that cerulenin resistance of Hcy-supplemented wildtype and the *sah1* mutant is not mediated by the inhibition of PL methylation. Furthermore, Hcy-supplemented wildtype and the *sah1* mutant displayed cerulenin resistance in the presence of inositol and choline (complete YPD medium), further indicating that neither choline nor inositol mediates cerulenin resistance of Hcy-supplemented wildtype and the *sah1* mutant.

**Figure 8.**
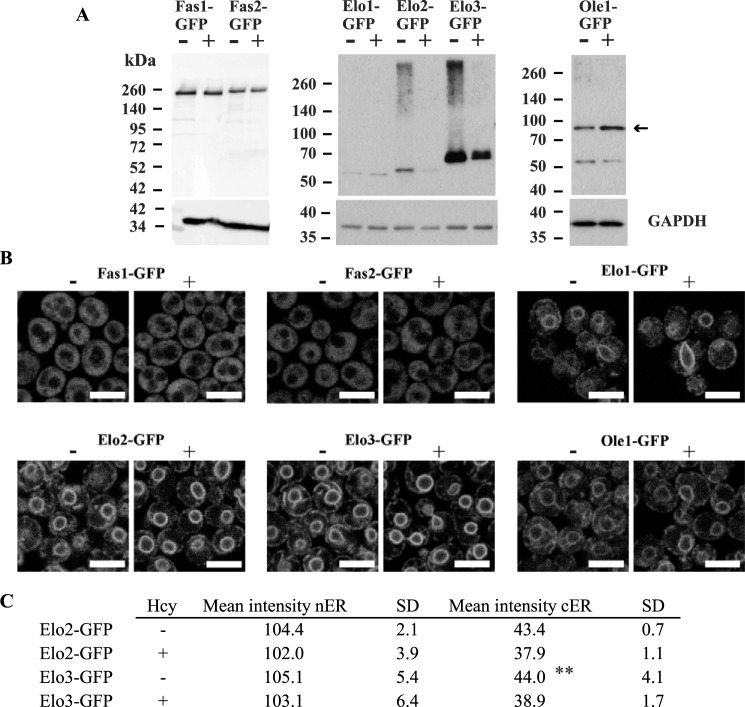
**Abundance and localization of GFP-tagged proteins involved in FA synthesis in yeast cultivated in the presence and absence of Hcy.** *A*, Fas1, Fas2, Elo1, Elo2, Elo3, and Ole1-GFP fusion protein levels. *B*, subcellular localization of Fas1, Fas2, Elo1, Elo2, Elo3, and Ole1-GFP fusion proteins. Logarithmic-phase yeast strains expressing C-terminally tagged GFP fusion proteins Fas1, Fas2, Elo1, Elo2, Elo3, and Ole1-GFP (EUROSCARF) were cultivated overnight in YPD medium, washed, shifted to −I/−C containing or not containing 2 mm Hcy, and cultivated for 6 h before analysis of GFP fusion protein levels by Western blotting (*A*) and GFP fusion protein localization by confocal laser-scanning microscopy (*B*). *C*, quantitation of Elo2-GFP and Elo3-GPF nuclear ER- and cortical ER-labeled structures. For details, see “Experimental procedures.” Data are mean ± S.D. (*error bars*) from more than 150 cells (*p* = 0.0019).

Although cerulenin resistance of wildtype cells grown in the presence of Hcy suggests an up-regulated FAS activity consistent with increased cellular FA content, this increase was not reflected in the amount of protein (Fig. 8, *A* and *B*), suggesting perhaps posttranslational modifications improving enzyme activity of an as yet unknown nature. Ole1 appears to be unaffected by the presence of Hcy (Fig. 8, *A* and *B*). In contrast, the abundance of Elo2- and Elo3-GFP fusion proteins was significantly attenuated in response to Hcy, as shown by Western blotting (Fig. 8*A*). Furthermore, analysis of subcellular localization of proteins involved in FA synthesis reveled that Elo2-GFP was significantly reduced at the cortical ER but not at the nuclear ER in response to Hcy (Fig. 8, *B* and *C*). The physiological relevance of this observation is currently unclear; however, the overall reduction of protein content indicates a reduced capacity of FA elongation in response to Hcy. This correlates with the observed changes in the FA chain length distribution in Hcy-supplemented wildtype and the *sah1* mutant (Figs. 3 and 4). A slight increase in the abundance of Ole1 (Fig. 8*A*), which also correlates with increased C16:1 content (Figs. 3 and 4), suggests an up-regulation of FA desaturation in response to Hcy in yeast. However, live cell fluorescence microscopy showed neither increased protein levels nor changes in protein localization of Ole1-GFP (Fig. 8, *A* and *B*). Taken together, the up-regulated FA and TG content, the shift in FA composition of cellular lipids, and the deregulation of the enzymes involved in FA synthesis indicate that Hcy regulates lipid metabolism in yeast. Suppression of dysregulated lipid metabolism in Hcy-supplemented wildtype by the AltPW shows that the deregulation of lipid metabolism in response to Hcy in yeast is due to AdoHcy accumulation. The dysregulation of FA synthesis in response to Hcy and AdoHcy further indicates that lipid metabolic enzymes beyond PL methyltransferases are highly susceptible to elevated Hcy and an accumulation of the product inhibitor of AdoMet-dependent methylation, AdoHcy.

## Discussion

An accumulation of Hcy and AdoHcy is linked to a number of pathological consequences and numerous disorders of modern society (6, 42–44). Because both metabolites are physiologically connected through the Sah1/AHCY-catalyzed reaction, an elevation of Hcy levels may lead to similar pathological consequences as an elevation of AdoHcy levels (4, 12–14). Both an accumulation of Hcy and an accumulation of AdoHcy are linked to the deregulation of lipid metabolism in mammals as well as in yeast (4, 7, 8, 18, 19, 23–26), suggesting that underlying mechanisms are related.

Here, we developed a yeast model of HHcy to identify mechanisms that dysregulate lipid metabolism. Hcy supplementation to wildtype yeast, similarly to AdoHcy accumulation in the *sah1* mutant, up-regulated cellular FA and TG content and induced a shift in FA composition (Figs. 3 and 4). Expression of the irreversible AltPW from *E. coli* enabled the dissection of AdoHcy- from Hcy-dependent effects and showed that indeed AdoHcy is the key trigger of the deregulation of lipid metabolism in response to Hcy in wildtype yeast (Fig. 6 and Figs. S1 and S2).

Defective PE methylation and concomitant inhibition of PC synthesis in the absence of choline may lead to a redirection of FA metabolic flux from membrane PL, particularly PC, toward storage TG. Indeed, mutants lacking the Cho2 and Opi3 PE- or PL-N-methyltransferases, respectively, similarly to Hcy-supplemented wildtype and the *sah1* mutant, display reduced PC and elevated TG content in the absence of choline (4). TG accumulation in the mutants deficient in PL methylation, however, is suppressed by the addition of choline, which channels any buildup of the TG precursor diacylglycerol (DG) into the synthesis of PC via the CDP-choline pathway (4). In contrast, choline supplementation of the yeast mutant depleted of Sah1, while restoring PC content, could not suppress TG accumulation (4), suggesting that mechanisms leading to TG accumulation in yeast deficient in PL methylation and AdoHcy hydrolysis differ. In line with this, we observed that the yeast *cho2opi3* mutant, which lacks both methyltransferases involved in PE-to-PC methylation, did not display similar resistance to cerulenin as Hcy-supplemented wildtype and the *sah1* mutant (Fig. 7), further indicating that the deregulation of lipid metabolism in response to Hcy/AdoHcy goes beyond deficient PL methylation.

Resistance of the *sah1* mutant and wildtype cells treated with Hcy to cerulenin (Fig. 7) suggests an up-regulation of FAS activity in response to Hcy/AdoHcy. Surprisingly, content of Fas1 and Fas2 subunits of FAS complex was unaltered, and no change in the cytosolic localization of FAS subunits in response to Hcy was observed (Fig. 8). In contrast, we observed alterations in protein levels of the condensing enzymes Elo2 and Elo3 of the FA elongase complex and Ole1, the sole FA desaturase, as well as a decrease of Elo2-GFP at the cortical ER in response to Hcy (Fig. 8, *B* and *C*). Whereas overall these alterations are consistent with the observed changes in FA profiles, the underlying molecular mechanisms affecting enzyme levels, activities, and/or localization in response to elevated Hcy are unclear. In mammals, FAS was reported to be phosphorylated (45) and acetylated (46), but there is no evidence available at present that protein methylation plays any role in regulating FAS activity in mammals or yeast. An involvement of methylation in the regulation of enzymatic activity was shown for protein phosphatase 2A; the C-terminal methylation of the Pph21/22 subunits of the protein phosphatase 2A regulates complex formation and enzymatic activity (47). However, whether the observed enzyme changes leading to deregulated lipid metabolism in response to Hcy/AdoHcy may be mediated by direct methylation defects or are a consequence of indirect effects (*e.g.* on (as yet unknown) kinases/phosphatases) still remains to be determined.

## Experimental procedures

### Strains, media, and growth conditions

*Saccharomyces cerevisiae* strains used in this study are congenic with BY4741, a derivative of S288C, and are listed in Table 1. Yeast strains expressing C-terminally tagged GFP full-length fusion proteins Fas1, Fas2, Elo1, Elo2, Elo3, and Ole1-GFP were obtained from EUROSCARF (48). Cells were grown at 30 °C in YPD medium containing 10 g/liter yeast extract, 20 g/liter peptone, and 20 g/liter glucose or in inositol- and choline-free synthetic minimal medium (−I/−C) containing 1.9 g/liter yeast nitrogen base without inositol, amino acids, and ammonium sulfate (Formedium), 5 g/liter ammonium sulfate, 20 g/liter glucose, supplemented with 20 mg/liter adenine, 20 mg/liter arginine, 20 mg/liter histidine, 60 mg/liter leucine, 230 mg/liter lysine, 20 mg/liter methionine, 20 mg/liter tryptophan, and 40 mg/liter uracil (49). Standard synthetic defined medium (SD), which contained 1.9 g/liter yeast nitrogen base without amino acids and ammonium sulfate (Formedium), 5 g/liter ammonium sulfate, 20 g/liter glucose, and amino acids as described above, differed from −I/−C only in that it contained 10 μm inositol. Inositol- and choline-containing medium (+I/+C) was the −I/−C medium containing 75 μm inositol and 1 mm choline. Sporulation medium contained 2.5 g/liter yeast extract, 1 g/liter glucose, and 10 g/liter potassium acetate. To select for the pYEX4T-1 (Clontech) plasmid, yeast transformants were grown on SD lacking uracil (SD−Ura). Induction of the *CUP1^p^* promoter was achieved by supplementation of media with 0.05 mm CuSO_4_. Media were solidified by the addition of 20 g/liter agar.

**Table 1:** **Strains used in this study**

Strain	Genotype	Source
WT	*MAT a his3*Δ*1 leu2*Δ*0 ura3*Δ*0*	This study
*met25*	*MAT a his3*Δ*1 leu2*Δ*0 ura3*Δ*0 met15*Δ*0*	This study
WT pYEX4T-1	*MAT a his3*Δ*1 leu2*Δ*0 ura3*Δ*0 pYEX4T-1*	This study
WT pYEX4T-1 [Pfs/LuxS]	*MAT a his3*Δ*1 leu2*Δ*0 ura3*Δ*0 pYEX4T-1* [*Pfs/LuxS*]	This study
*sah1/SAH1*	*MAT a/a his3*Δ*1/his3*Δ*1 leu2*Δ*0/leu2*Δ*0 ura3*Δ*0/ura3*Δ*0 LYS2/lys2*Δ*0 met25*Δ*0/MET25 sah1::kanMX4/SAH1*	Euroscarf
*sah1*	*MAT a his3*Δ*1 leu2*Δ*0 ura3*Δ*0 sah1::kanMX4*	This study
*sah1* pYEX4T-1	*MAT a his3*Δ*1 leu2*Δ*0 ura3*Δ*0 sah1::kanMX4 pYEX4T-1*	This study
*sah1* pYEX4T-1 [Pfs/LuxS]	*MAT a his3*Δ*1 leu2*Δ*0 ura3*Δ*0 sah1::kanMX4 pYEX4T-1* [*Pfs/LuxS*]	This study
*sah1met25*	*MAT a his3*Δ*1 leu2*Δ*0 ura3*Δ*0 met15*Δ*0 sah1::kanMX4*	This study
*cho2opi3*	*MAT a his3*Δ*1 leu2*Δ*0 ura3*Δ*0 cho2::kanMX4 opi3::natMX4*	This study
Fas1-GFP	*MAT a his3*Δ*1 leu2*Δ*0 met15*Δ*0 ura3*Δ*0 Fas1-GFP-HIS3MX6*	Euroscarf
Fas2-GFP	*MAT a his3*Δ*1 leu2*Δ*0 met15*Δ*0 ura3*Δ*0 Fas2-GFP-HIS3MX6*	Eurascarf
Elo1-GFP	*MAT a his3*Δ*1 leu2*Δ*0 met15*Δ*0 ura3*Δ*0 Elo1-GFP-HIS3MX6*	Euroscarf
Elo2-GFP	*MAT a his3*Δ*1 leu2*Δ*0 met15*Δ*0 ura3*Δ*0 Elo2-GFP-HIS3MX6*	Euroscarf
Elo3-GFP	*MAT a his3*Δ*1 leu2*Δ*0 met15*Δ*0 ura3*Δ*0 Elo3-GFP-HIS3MX6*	Euroscarf
Ole1-GFP	*MAT a his3*Δ*1 leu2*Δ*0 met15*Δ*0 ura3*Δ*0 Ole1-GFP-HIS3MX6*	Euroscarf

### Cloning of genes encoding E. coli enzymes Pfs and LuxS

A 699-base pair fragment containing the *pfs* gene encoding *S*-adenosylhomocysteine nucleosidase (EC 3.2.2.9) was amplified from *E. coli* XL-1 Blue genomic DNA (Stratagene^TM^) by PCR using 5′-TAGTAGGATCCATGAAAATCGGCATCATTGG-3′ and 5′-TGTATGAATTCTTAGCCATGTGCAAGTTTCTG-3′ forward and reverse primers, respectively, and Pwo^super yield^ DNA polymerase (Roche Applied Science). A 516-base pair fragment containing the *luxS* gene encoding *S*-ribosylhomocysteine lyase (EC 4.4.1.21) was amplified from *E. coli* K12 genomic DNA by PCR using 5′-TCTGAGAATTCATGCCGTTGTTAGATAGCTTCAC-3′ and 5′-TATTCTCGAGCTAGATGTGCAGTTCCTGCAACTT-3′ forward and reverse primers, respectively. The *pfs* fragment was cloned into the BamHI and EcoRI restriction sites of the multiple cloning site of the pYEX4T-1 vector yielding the plasmid pYEX4T-1 [Pfs]. The *luxS* fragment was cloned into the EcoRI and XhoI restrictions sites of the multiple cloning site of pYEX4T-1 yielding the plasmid pYEX4T-1 [LuxS]. For the construction of a vector containing both GST-tagged *pfs* and *luxS* genes, the whole expression cassette for the *pfs* gene was amplified from pYEX4T-1 [Pfs] by PCR using 5′-TATTTTGACGTCCCAAGCTTGCATGTCTTTTGCT-3′ and 5′-TAAATTGACGTCCATCGCGGTACCATGAATAGCTT-3′ primers and inserted into the AatII restriction site of the pYEX4T-1 [LuxS] plasmid. The plasmids pYEX4T-1 [Pfs], pYEX4T-1 [LuxS], and pYEX4T-1 [Pfs/LuxS] were verified for correct insertion by restriction analysis and by sequencing.

### Expression of N-terminally GST-tagged E. coli enzymes Pfs and LuxS in yeast

Yeast wildtype and *sah1* mutant cells were transformed by the method of Gietz *et al.* (50). Transformants were selected for uracil prototrophy on SD−Ura medium. To detect expression of the GST fusion proteins, transformants were inoculated from overnight SD−Ura cultures in 5 ml of SD−Ura containing or not 0.05 mm CuSO_4_ at *A*_600_ = 1. Cells were collected after cultures reached *A*_600_ = 10, and whole-yeast cell extracts were prepared by the method of Baerends *et al.* (51). Proteins were separated on 10% SDS-polyacrylamide gels and transferred to a nitrocellulose membrane (Bio-Rad). Goat anti-GST antibody (Sigma) followed by horseradish peroxidase-conjugated anti-goat IgG (Pierce) secondary antibody was used to detect GST fusion proteins. Chemiluminescent Western blotting substrate (Thermo Scientific) was used for detection.

### AdoHcy quantification

AdoHcy was extracted by the method of Gellekink (52) with minor modifications. Yeast were grown overnight in SD−Ura, rediluted in the same medium to *A*_600_ = 0.03, grown for an additional 24 h, and reinoculated in the same medium to *A*_600_ = 0.3. After 6 h of growth, 100 *A*_600_ units were collected, stored at −70 °C, or processed immediately. For AdoHcy extraction, cells were resuspended in 5 ml of cold 0.091 m acetic acid and broken with 5-ml glass beads in a Merckenschlager homogenizer (Braun Biotech International) under CO_2_ cooling in the presence of 15 nmol of AdoHcy-*d*_4_ (CDN Isotopes) as an internal standard (ISTD). Glass beads were separated from the lysate by centrifugation and washed twice with 0.091 m cold acetic acid, and the supernatants were combined. AdoHcy and the internal standard were extracted from the cell lysates by solid-phase extraction on Bond Elut PBA columns (Varian Inc.) (52). Chromatographic separation of AdoHcy was performed using an AQUITY-UPLC® system (Waters) on a BEH-C18 column, 1.7-μm particle size, 3 × 150 mm (Waters). The gradient started at 96% solvent A (0.1% formic acid in water) and increased to 100% solvent B (methanol, 0.1% formic acid) over 9 min at a flow rate of 100 μl/min. AdoHcy was measured in positive ESI mode on a SYNAPT^TM^ G1 qTOF HD mass spectrometer (Waters). The extracted ion chromatograms of *m*/*z* 385 (AdoHcy) and *m*/*z* 389 (AdoHcy-*d*_4_) were used for peak integration.

### Lipid analysis

Yeast wildtype or the *sah1* mutant cells were grown overnight in +I/+C, washed, inoculated to *A*_600_ = 0.3 in −I/−C containing or not 1 mm Hcy, and cultivated for 6 h. Afterward, 20 *A*_600_ units were collected, stored at −70 °C, or processed immediately. Yeast expressing the AltPW were cultivated as described under “AdoHcy quantification.” Cells were resuspended in 5 ml of chloroform/methanol (2:1, v/v) (CM), and 50 μl of 0.05 mg/ml lipid ISTD mix (53) were added. Cells were disrupted in the presence of glass beads in a Heidolph Multi Reax test tube shaker at 4 °C for 30 min. Lipids were extracted according to the Folch method (54). For analysis in positive ESI mode, dried total lipid extracts were dissolved in 1 ml of CM and diluted with 2-propanol (1:5, v/v). For analysis in negative ESI mode, dried total lipid extracts were dissolved in 1 ml of CM/2-propanol (1:10, v/v). Chromatographic separation was performed using an AQUITY-UPLC system equipped with a BEH-C18 column, 2.1 × 150 mm, 1.7 μm (Waters). A binary gradient was applied; solvent A consisted of water/methanol (1:1, v/v), and solvent B was 2-propanol. Both solvents contained phosphoric acid (8 μm), ammonium acetate (10 mm), and formic acid (0.1 volume %). The linear gradient started at 45% solvent B and increased to 100% solvent B within 32 min, and the flow rate was 150 μl/min. The column compartment was kept at 50 °C. A SYNAPT^TM^ G1 qTOF HD mass spectrometer equipped with an ESI source was used for analysis. The following source parameters were used: capillary temperature, 100 °C; desolvatization temperature, 400 °C; N_2_ as nebulizer gas. The capillary voltage was 2.6 kV in positive and 2.1 kV in negative ionization mode. Leucine-enkephaline ([MH^+^]: *m*/*z* 556.2771 and [M^−^H^−^]: *m*/*z* 554.2615) was used as reference substance in the lock-spray. Data acquisition was done by MassLynx version 4.1 software (Waters). For lipid analysis, Lipid Data Analyzer version 1.6.2 software was used (55). Data were normalized (analyte/ISTD) for recovery, extraction, and ionization efficacy using ISTDs.

### FA analysis

Dried lipid extracts were resuspended in 1 ml of CM. A 200-μl aliquot of the extract was transferred to a Pyrex glass tube, and 100 μl of ISTD (C17:0 FA, 250 μm in methanol) were added. FAs were transesterified using 2 ml of 2% HCl in methanol and 0.5 ml of toluene. After a 1-h incubation at 100 °C, 1 ml of ice-cold water was added, and methyl esters were extracted twice with 2 ml of hexane. The samples were dried under a stream of nitrogen, resuspended in 100 μl of hexane, and transferred to autosampler vials. GC-FID was performed using a Trace GC (Thermo Scientific). 1 μl of sample solution was injected (injector temperature 250 °C) and separated on a 25-m column (CP-FFAP CB, ID 0.32 mm, 0.3-μm film) using a temperature gradient from 110 to 300 °C. Data analysis was performed using XCalibur version 1.4 software (Thermo Scientific). GC-MS was performed using the Trace GC coupled to a DSQ single-stage quadrupole mass spectrometer (Thermo Scientific). 1 μl of sample solution was injected (injector temperature 250 °C) and separated on a 60-m column (CP-FFAP CB, ID 0.25 mm, 0.25 μm film) using a temperature gradient from 110 to 300 °C. Data analysis was performed using XCalibur version 1.4 software. Calibration for FA C16:0/1 and C18:0/1 was performed between 1 and 133 μg/ml, C17:0 FA was used as internal standard.

### Western blotting

Yeast strains expressing chromosomally C-terminally tagged GFP fusion proteins Fas1, Fas2, Elo1, Elo2, Elo3, and Ole1-GFP were grown overnight in YPD, washed, inoculated at *A*_600_ = 0.25 in −I/−C in the presence or absence of 2 mm Hcy, and cultivated for an additional 6 h. 3 *A*_600_ units of logarithmically growing cells were harvested, and whole-yeast cell extracts were prepared by the method of Horvath and Riezman (56). Proteins were resolved on 10% SDS-polyacrylamide gels and blotted onto nitrocellulose membranes (Bio-Rad). Mouse monoclonal anti-GFP antibody (Roche Applied Science) followed by horseradish peroxidase–conjugated anti-mouse IgG (Pierce) secondary antibody was used to detect GFP fusion proteins. Rabbit polyclonal anti-GAPDH antibody (57) followed by horseradish peroxidase–conjugated anti-rabbit IgG (Pierce) secondary antibody was used to detect GAPDH, which served as loading control. Chemiluminescent Western blotting substrate (Thermo Scientific) was used for detection.

### Confocal laser-scanning microscopy

Imaging was performed using a Leica SP5 confocal microscope (Leica Inc.) with spectral detection and a HCX PL APO ×63 numeric aperture 1.4 oil immersion objective. GFP was excited at 488 nm, and emission was detected between 500–550 nm. Fluorescence signal was detected using a hybrid photon detector. Fluorescence and transmission images were acquired simultaneously.

### Image processing

Image processing and quantification of fluorescent protein signal were performed using the open-source software Fiji (58). Initially, image noise was reduced by Gaussian filtering (σ value 1). Ringlike nER structures typically showing increased signal compared with cER structures were extracted from the data sets using the autothreshold/triangle method. False-segmented structures located at the cER were manually removed from the binary data. Not entirely registered nER structures were closed using the “close” binary function. The mean gray-level intensity of segmented nER structures was computed. All segmented nER structures were selected using the “create selection” function, and selections were filled with the background color (*black*) using the “fill” function. Afterward, cER structures were extracted from the fluorescence data sets using the autothreshold/entropy method while excluding the selections previously created from the segmentation process. Again, the mean gray-level intensity of extracted cER structures was computed.

### Statistics

Data are shown as the means ± S.D. Statistical significance between two groups was determined by unpaired Student's two-tailed *t* test. The following levels of statistical significance were used: *, *p* < 0.05; **, *p* < 0.01; ***, *p* < 0.001.

## Author contributions

M. V. constructed all strains used in this study; M. V., M. R., T. O. E., G. N. R., and O. T. performed lipid analysis; M. V. and M. R. performed plate tests; H. W. performed microscopy and image analysis; M. V. and M. R. performed Western blotting; S. S. and N. M. cloned and functionally expressed the AltPW in yeast; M. V. performed AdoHcy analysis; O. T. designed the study and wrote the paper. All authors reviewed the results and approved the final version of the manuscript.

## Supplementary Material

Supporting Information

## Footnotes

2 The abbreviations used are:

AdoMet: *S*-adenosyl-l-methionine
AHCY: *S*-adenosyl-l-homocysteine hydrolase in mammals
Sah1: *S*-adenosyl-l-homocysteine hydrolase in yeast
AdoHcy: *S*-adenosyl-l-homocysteine
Hcy: homocysteine
HHcy: hyperhomocysteinemia
FA: fatty acid
TG: triacylglycerol
PL: phospholipid
Pfs: *S*-adenosylhomocysteine nucleosidase
LuxS: *S*-ribosylhomocysteine lyase
AltPW: alternative pathway for AdoHcy catabolism
PC: phosphatidylcholine
PE: phosphatidylethanolamine
FAS: FA synthase
GST: glutathione *S*-transferase
GFP: green fluorescent protein
DG: diacylglycerol
ISTD: internal standard
CM: chloroform/methanol
SD: synthetic defined
ER: endoplasmic reticulum
GAPDH: glyceraldehyde-3-phosphate dehydrogenase.
