# Supplementary material for: Homocysteine regulates fatty acid and lipid metabolism in yeast
Source: J Biol Chem. 2018 Feb 6;293(15):5544–55. doi: 10.1074/jbc.M117.809236 (PMC5900771; doi:10.1074/jbc.M117.809236)
Supplement: Supporting Information [file supp_293_15_5544__index.html]

Homocysteine regulates fatty acid and lipid metabolism in yeast — Homocysteine regulates fatty acid and lipid metabolism in yeast — Homocysteine regulates lipid metabolism — Supporting Information 

# Homocysteine regulates fatty acid and lipid metabolism in yeast

## Supporting Information

- Supplemental figures S1 and S2 (.pdf, 210 KB) - Expression of the AltPW suppresses the deregulation of lipid metabolism in the yeast sah1 mutant and Hcy-supplemented wild type
