# Supplementary material for: Homocysteine regulates fatty acid and lipid metabolism in yeast
Source: J Biol Chem. 2018 Feb 6;293(15):5544–55. doi: 10.1074/jbc.M117.809236 (PMC5900771; doi:10.1074/jbc.M117.809236)
Supplement: Supporting Information [file 10.1074_M117.809236_jbc.M117.809236-1.pdf]

Homocysteine regulates fatty acid and lipid metabolism in yeast

Myriam Visram[1], Maja Radulovic[1], Sabine Steiner[1], Nermina Malanovic[1], Thomas O. Eichmann[1], Heimo Wolinski[1], Gerald N. Rechberger[1,2] and Oksana Tehlivets[1]*

Figure 1S. Expression of the AltPW suppresses the deregulation of lipid metabolism in the yeast *sah1* mutant.

Figure 2S. Expression of the AltPW suppresses the deregulation of lipid metabolism in Hcy-supplemented wild type yeast.

Figure S1

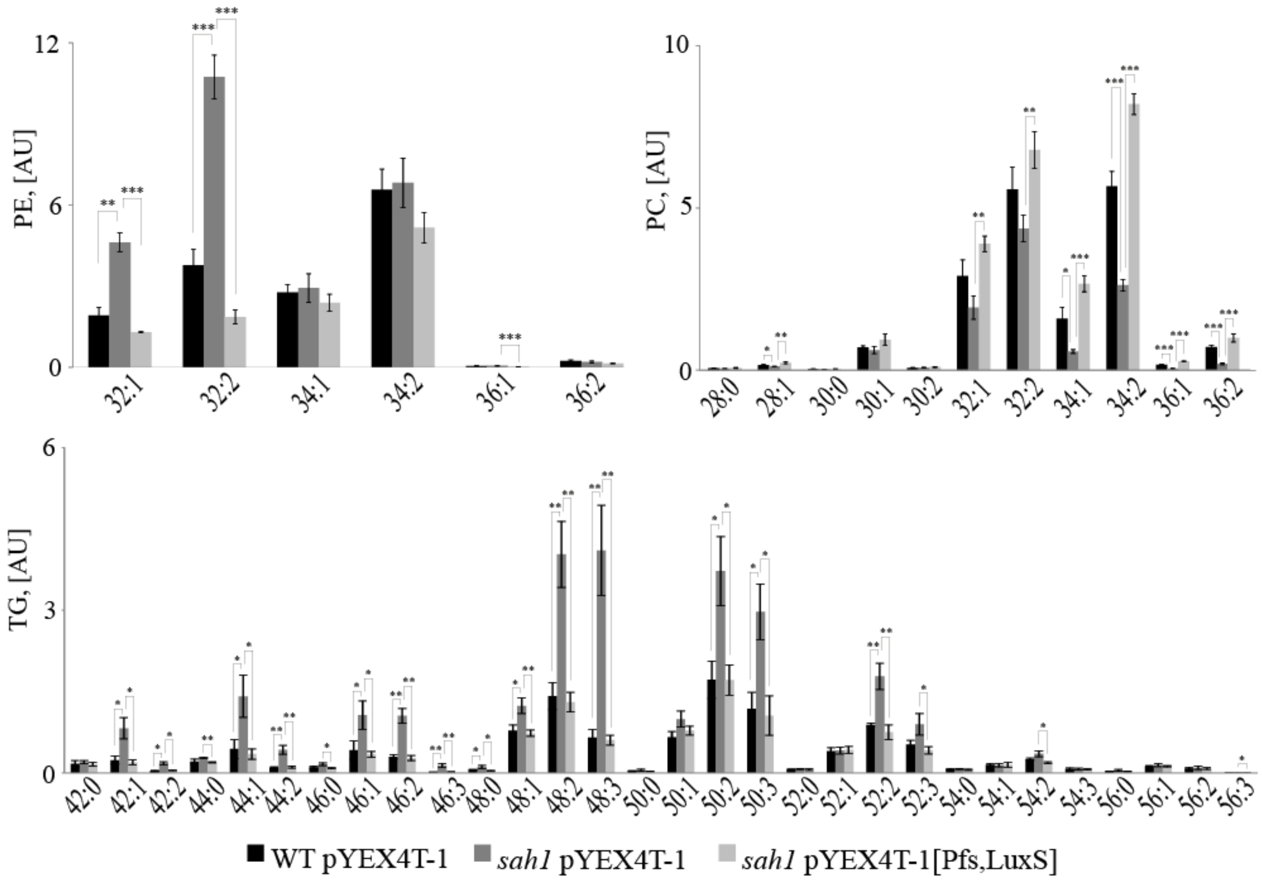

Figure 1S. Expression of the AltPW suppresses the deregulation of lipid metabolism in the yeast *sah1* mutant. Species distribution of PE, PC and TG. Logarithmic-phase wild type and the *sah1* mutant expressing the AltPW or carrying the empty plasmid were cultivated in SD-Ura medium, reinoculated in the same medium supplemented or not with 0.05 mM $CuSO_4$ and cultivated for 6 h before lipid extraction. TG, PE and PC were normalized to ISTDs. Data only for strains grown in the absence of additional $CuSO_4$ supplementation are shown. Data are mean ± SD from three independent experiments (***, $p < 0.001$; **, $p < 0.01$; *, $p < 0.05$).

Figure S2

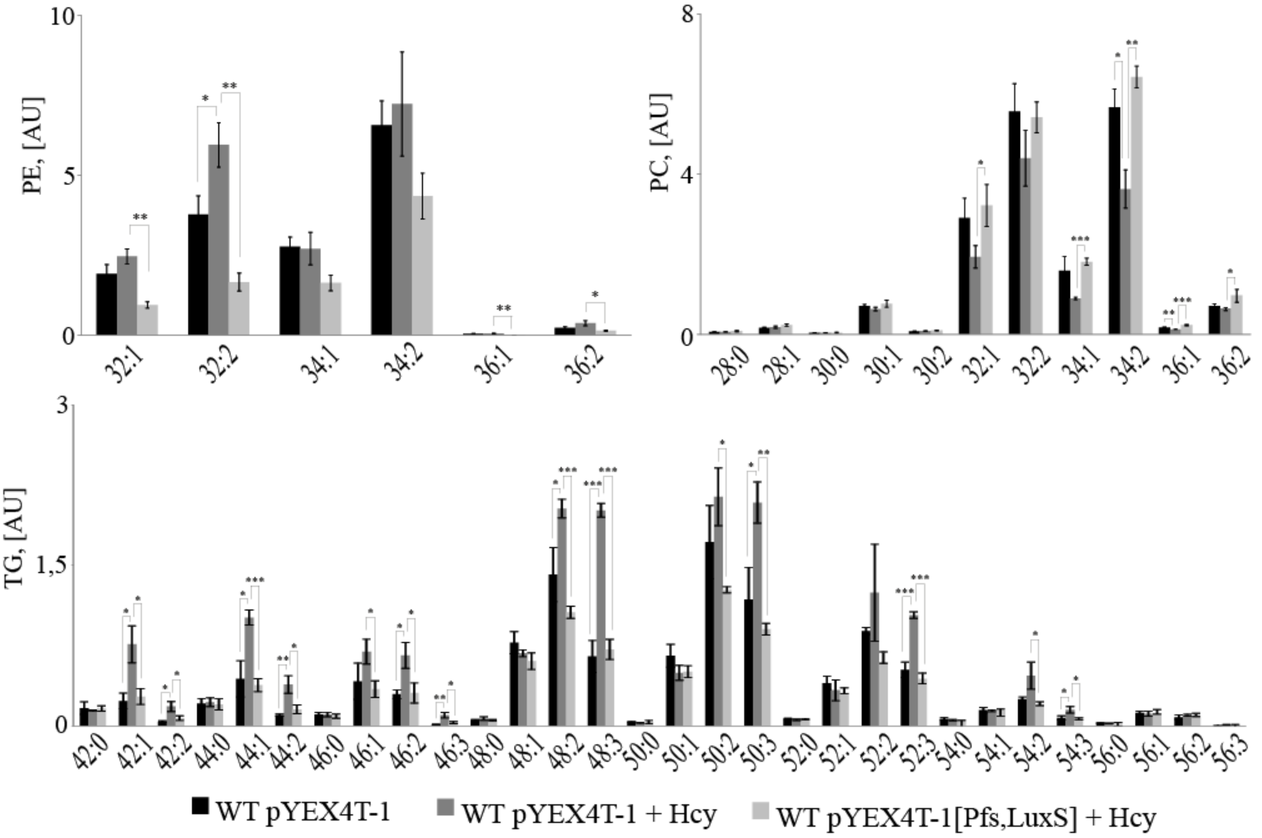

Figure 2S. Expression of the AltPW suppresses the deregulation of lipid metabolism in Hcy-supplemented wild type yeast. Species distribution of PE, PC and TG. Logarithmic-phase wild type expressing the AltPW or carrying the empty plasmid was cultivated in SD-Ura medium, reinoculated into same medium supplemented or not with 1 mM Hcy in the absence of additional $CuSO_4$ supplementation and cultivated for 6 h before lipid extraction. TG, PE and PC were normalized to ISTDs. Data are mean ± SD from three independent experiments (***, $p < 0.001$; **, $p < 0.01$; *, $p < 0.05$).
